# TAE226, a dual inhibitor of focal adhesion kinase and insulin‐like growth factor‐I receptor, is effective for Ewing sarcoma

**DOI:** 10.1002/cam4.2647

**Published:** 2019-11-06

**Authors:** Hiroshi Moritake, Yusuke Saito, Daisuke Sawa, Naoki Sameshima, Ai Yamada, Mariko Kinoshita, Sachiyo Kamimura, Takao Konomoto, Hiroyuki Nunoi

**Affiliations:** ^1^ Division of Pediatrics Faculty of Medicine University of Miyazaki Miyazaki Japan; ^2^ Department of Pathology Faculty of Medicine University of Miyazaki Miyazaki Japan

**Keywords:** Ewing sarcoma, focal adhesion kinase, insulin‐like growth factor‐I receptor, metastasis, TAE226

## Abstract

The outcomes for relapsed and metastatic Ewing sarcoma (EWS) is extremely poor. Therefore, it is important to identify the tumor‐specific targets in these intractable diseases. High focal adhesion kinase (FAK) transcript expression levels in EWS cell lines are known. TAE226 is a dual inhibitor of FAK and insulin‐like growth factor‐I receptor (IGF‐IR), while PF‐562,271 is a dual inhibitor of FAK and proline‐rich tyrosine kinase 2. We compared the cytotoxicity of TAE226 and PF‐562,271 toward three EWS cell lines. TAE226 strongly inhibited proliferation of three cell lines when compared with PF‐562,271. Furthermore, we investigated the efficacy of TAE226 as well as its mechanism of action against EWS. A stable EWS cell line with FAK and IGF‐IR knocked down was established, and microarray analysis revealed dysregulated expression in various pathways. TAE226 treatment of EWS cell lines induced cell cycle arrest, apoptosis, AKT dephosphorylation, and inhibition of invasion. We demonstrated that TAE226 drastically inhibits the local growth of primary tumors and metastasis in EWS using mouse models. Furthermore, the combination of TAE226 and conventional chemotherapy proved to exert synergistic effects. TAE226 may be a candidate single agent or combined therapy drug to be developed for patients who have relapse and metastatic EWS tumors in future.

## Graphical abstract

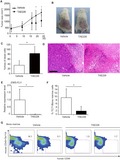

## 1 INTRODUCTION

Ewing sarcoma (EWS) is an aggressive tumor typically arising from bone. Peak incidence occurs during adolescence with second and third decade. It is characterized by highly recurrent translocation involving *EWS* genes, such as *EWS‐FLI1* and *EWS‐ERG*.^1^, ^2^ New therapeutic strategies for the treatment of EWS are essential, because intensive multimodal therapy cannot successfully treat the majority of patients with metastases or relapse.^3^, ^4^ Moreover, long‐term morbidity is significant in children who are cured after intensive treatment.^5^

Targeting aberrantly activated tyrosine kinases and tumor‐specific antigens is an attractive treatment approach with recent success in several types of malignancies. Selective kinase inhibitors have been developed and their efficacies in patients with *BCR/ABL*‐positive chronic myeloid leukemia,^6^ epidermal growth factor receptor (*EGFR*)‐mutated lung cancer,^7^ and *KIT*‐mutated gastrointestinal stromal tumors.^8^ In pediatric solid tumors, clinical trials testing the anaplastic lymphoma kinase (ALK)‐inhibitor crizotinib for neuroblastoma with *ALK* mutations resulted in little effect, although objective activity was recognized in anaplastic large cell lymphoma with *NPM/ALK* mutations.^9^ In EWS, clinical trials using insulin‐like growth factor‐I receptor (IGF‐IR)‐targeting antibodies induced clinical responses in a subset of patients with EWS.^10^

Focal adhesion kinase (FAK) is a non‐receptor tyrosine kinase and central regulator of integrin signaling that mediates many cellular functions, including adhesion, migration, growth‐factor signaling, proliferation, and survival. FAK activation is initiated through a variety of extracellular signals that allow the cell to adapt to changes in the surrounding environment.^11^, ^12^ Aberrant FAK activation is a frequent event in cancer, promoting cell growth, survival, and invasion.^13^, ^14^, ^15^ Previously, we reported recognition of high FAK transcript expression levels in EWS cell lines compared with normal fibroblasts and other kinds of pediatric solid tumor cell lines.^16^ Crompton et al identified FAK as a candidate therapeutic target through high‐throughput tyrosine kinase activity profiling.^17^ They investigated the efficacy of PF‐562,271, a dual tyrosine kinase inhibitor of FAK and proline‐rich tyrosine kinase 2 (PYK2) that induced apoptosis and downregulation of AKT/mammalian target of rapamycin (mTOR) as well as Crkl‐associated substrate activity in EWS. TAE226 is another dual tyrosine kinase inhibitor of FAK and IGF‐IR that has been shown to have potent anticancer effects in several types of malignancies.^18^, ^19^, ^20^, ^21^, ^22^, ^23^, ^24^, ^25^, ^26^ Considering the relationship between IGF‐IR and EWS, TAE226 could possibly be superior to PF‐562,271 as a candidate drug for EWS treatment. In the present study, we compared the cytotoxicity of both TAE226 and PF‐562,271 toward multiple EWS cell lines. Furthermore, we investigated the efficacy of TAE226 in vitro and in vivo, as well as its mechanism of action against EWS.

## 2 MATERIALS AND METHODS

### 2.1 Reagents

TAE226 and PF‐562,271 were kindly provided by Novartis Pharm AG and Pfizer, respectively. The primary antibodies to the following proteins were purchased from Cell Signaling Technology and used for western blotting: FAK, IGF‐IRb, phospho‐IGF‐IRb (Tyr1135/1136), AKT, phospho‐AKT (Ser473), mTOR, phospho‐mTOR (Ser2448), S6 ribosomal protein, phosphor‐S6 ribosomal protein (Ser235/236), and cleaved poly‐ADP‐ribose polymerase (PARP). The phospho‐FAK (Tyr397) antibody was purchased from BD Pharmingen. An anti‐β‐actin antibody was used as a loading control. Recombinant human EGF, brain‐derived neurotrophic factor (BDNF), and transforming growth factor‐β (TGF‐β) were purchased from PeproTech. Recombinant human IGF‐I was purchased from Sigma‐Aldrich, and IGF‐IR inhibitor (picropodophyllin) was purchased from Santa Cruz Biotechnology.

### 2.2 Cell culture

TC71, SK‐ES‐1, RD‐ES, RD, and human epithelial kidney 293T cell lines were provided by the laboratory of Professor Tohru Sugimoto (University of Miyazaki, Kyoto Prefectural University of Medicine). The SU‐CCS‐1 cell line was kindly supplied by Dr Alan L. Epstein (Keck School of Medicine, University of Southern California), and the HS‐Os‐1 cell line was obtained from Riken Bioresource Center (Tsukuba, Japan). Human fibroblasts were obtained through the culture of mononuclear cells isolated from the bone marrow of healthy volunteer with informed consent. Except for HS‐Os‐1 and 293T, all cells were grown in RPMI (Sigma‐Aldrich) with 10% fetal bovine serum (Sigma‐Aldrich), 100 U/mL penicillin, and 100 µg/mL streptomycin (Nacalai Tesque Inc). HS‐Os‐1 and 293T cells were maintained in Dulbecco's Modified Eagle's Medium (Sigma‐Aldrich) with 10% fetal bovine serum (Sigma‐Aldrich), 100 U/mL penicillin and 100 µg/mL streptomycin (Nacalai Tesque Inc). SK‐ES‐1 and RD‐ES were cultured on collagen‐coated dishes or flasks.

### 2.3 Drug sensitivity of cell lines by 3‐(4,5‐dimethylthiazol‐2‐yl)‐2,5‐diphenyl tetrazolium bromide (MTT) assay

Drug sensitivity was determined by MTT assay using Cell Proliferation Kit I (Roche). Each cell line as well as human fibroblasts were seeded (5000 cells/well) onto 96‐well plates in drug‐free medium. The next morning, the medium in all wells was replaced with the medium containing different concentrations of TAE226, PF‐562,271, vincristine (Kyowa Hakko Kirin Co., Ltd.), doxorubicin (Kyowa Hakko Kirin Co.), etoposide (Bristol‐Myers Squibb), IGF‐IR or DMSO (control) and incubated for 48 hours. The antitumor effects of the reagents on each cell line were calculated in terms of 50% inhibitory concentration (IC_50_) values.

### 2.4 Western blotting

TC71 cells were cultured in 10‐mm dishes overnight and then treated with DMSO or different concentrations of TAE226 for 24 hours. Whole cells were lysed in 1 × cell lysis buffer (Pierce) supplemented with protease inhibitors (Roche) to extract proteins after washing with cold phosphate‐buffered saline. Equal amounts of total protein (20 μg) were separated by sodium dodecyl sulfate‐polyacrylamide gel electrophoresis and transferred to polyvinylidene difluoride membranes. The proteins on membranes were incubated overnight at 4°C with the primary antibodies. The following secondary antibodies were used: goat, antirabbit or antimouse immunoglobulin G‐conjugated horseradish peroxidase (Santa Cruz Biotechnology). To assess the effect of TAE226 on IGF‐IR under ligand‐free conditions, IGF‐IR phosphorylation after serum starvation followed by TAE226 treatment was analyzed. TC71 cells were serum‐starved for 3 hours before being treated with different concentrations of TAE226 for 2 hours. Then, they were stimulated by 100 ng/mL of recombinant IGF‐I (Sigma‐Aldrich) for 15 minutes prior to western blotting.

### 2.5 SelectScreen kinase profiling

IC_50_ values for FAK, IGF‐IR, AKT, and mTOR inhibition were calculated using the SelectScreen™ Kinase Profiling Service of Thermo‐Fisher Scientific (Madison, WI, USA). For each kinase, IC_50_ was calculated based on a 10‐point concentration curve of the test article and converted to Ki values. The protocols are available at the Thermo‐Fisher Scientific website (https://www.thermofisher.com/jp/ja/home/products-and-services/services/custom-services/screening-and-profiling-services/selectscreen-profiling-service/selectscreen-kinase-profiling-service.html).

### 2.6 Small hairpin RNA (shRNA) transfection

A vector expressing the shRNA of FAK (SHCLING‐NM_005607) was purchased from Sigma. They included five different clones (TRCN0000196310, TRCN0000194984, TRCN0000121318, TRCN0000121207, and TRCN0000121209) to inhibit expression of FAK. For the construction of vectors expressing shRNA of IGF‐IR, three oligos were prepared with the following sequences: IGF‐IR‐1, 5′‐CCGGGCCTTTCACATTGTACCGCATCTCGAGATGCGGTACAATGTGAAAGGCTTTTTG‐3′ (forward) and 5′‐AATTCAAAAAGCCTTTCACATTGTACCGCATCTCGAGATGCGGTACAATGTGAAAGGC‐3′ (reverse); IGF‐IR‐2, 5′‐CCGGGCCGAAGATTTCACAGTCAAACTCGAGTTTGACTGTGAAATCTTCGGCTTTTTG‐3′ (forward) and 5′‐AATTCAAAAAGCCGAAGATTTCACAGTCAAACTCGAGTTTGACTGTGAAATCTTCGGC‐3′ (reverse); IGF‐IR‐3, 5′‐CCGGCGGCAACCTGAGTTACTACATCTCGAGATGTACTAACTCAGGTTGCCGTTTTTG‐3′ (forward) and 5′‐AATTCAAAAACGGCAACCTGAGTTACTACATCTCGAGATGTAGTAACTCAGGTTGCCG‐3′ (reverse).

They were subcloned into pLKO.1 hygro (Addgene). Human epithelial kidney 293T cells were transfected with helper retrovirus and retroviral plasmids using FuGENE 6 Transfection Reagent (Promega). Retroviruses expressing shRNA were harvested 24‐60 hours after transfection and stored on ice.^27^ The transfected TC71 cells bearing the lowest FAK and IGF‐IR expression by real‐time quantitative reverse transcription‐polymerase chain reaction (RQ‐RT‐PCR) and western blotting were used for gene expression analysis.

### 2.7 RQ‐RT‐PCR

THUNDERBIRD SYBR qPCR Mix (Toyobo Co.) was used to evaluate the levels of EWS‐FLI1, FAK, and IGF‐IR expression. The primers used for RQ‐RT‐PCR were as follows: EWS‐FLI1, 5ʹ‐CCTACAGCCAAGCTCCAAGTC‐3ʹ, and 5ʹ ‐CTTACTGATCGTTTGTGCC‐3ʹ; FAK, 5ʹ ‐GAGGGTGTCAAGCCATGGAG‐3ʹ, and 5ʹ ‐GGCCCGTCACATTCTCGTAC‐3ʹ; IGF‐IR, 5ʹ ‐CCTGTGAAAGTGACGTCCTG‐3ʹ, and 5ʹ‐GGTGCTTCCTTGTAGTAAACG‐3ʹ. The copy number was normalized to GAPDH (GAPDH primers: 5ʹ ‐GAAGGTGAAGGTCGGAGT‐3ʹ and 5ʹ ‐GAAGATGGTGATGGGATTTC‐3ʹ).

### 2.8 Gene expression analysis

To identify disrupted pathways when FAK and IGF‐IR expression were knocked down in TC71 cells, gene expression assays were performed. Moreover, we also analyzed gene expression in TC71 cells treated with 10 μmol/L TAE226 for 6 hours. Preparation of cRNA hybridization and scanning of probe arrays for each sample was performed according to the manufacturer's protocols (Affymetrix) using a Cogentech Affymetrix microarray unit (Campus IFOM IEO) and a Human Genome U133A 2.0 Gene Chip (Affymetrix). The different gene expression patterns were analyzed using Gene Spring software version 12.1 (Agilent Technologies). Statistically significant genes were selected for final consideration when their expression was at least 2.0‐fold different in the test sample vs. the control. Genes that passed both the *P*‐value and fold change restriction thresholds were submitted for functional classification according to Gene Ontology annotations.

### 2.9 AKT phosphorylation by EGF, BDNF, and TGF‐β stimulation following TAE226 blockade

AKT phosphorylation after serum starvation followed by TAE226 treatment was examined to assess whether TAE226 suppresses EGF, BDNF, and TGF‐β signaling pathways in EWS. TC71 cells were seeded (1 million cells/well) onto 6‐well plates in a drug‐free medium. The next morning, the medium in all wells was replaced with the serum‐free medium for 3 hours. Subsequently, the cells were stimulated with 10 ng/mL EGF, 50 ng/mL BDNF, or 5ng/ml TGF‐β for 10 minutes after incubating with different concentrations of TAE226 for 2 hours. AKT phosphorylation was assessed using western blotting.

### 2.10 Cell cycle analysis

BrdU (10 μmol/L final concentration) was added to the culture medium, cells were incubated for 40 minutes, fixed, and stained according to the protocol of the BD Biosciences APC BrdU Flow kit, and analyzed using flow cytometry.

### 2.11 Apoptosis analysis

Cells were incubated with TAE226 in complete medium for 24 hours. Then, cells were washed, resuspended in 500 µL of binding buffer, and incubated with 5 µL of APC Annexin‐V (BioLegend) and 2 µg/mL of 4',6‐diamidino‐2‐phenylindole dihydrochloride for 15 minutes. Samples were analyzed using a JSAN cell sorter (Bay Bioscience).

### 2.12 Cellular invasion assay

Invasion assays were performed in modified Boyden chambers (Chemotaxis 8μ; Kurabo); cells (2 × 10^5^) in 300 μL of serum‐free RPMI were loaded into the upper well. RPMI (500 μL) with 10% fetal bovine serum was added to the lower well. After a 24‐hours incubation, cells in the upper chambers were removed with a cotton swab, and the filter was fixed and stained with *Diff*‐*Quik* stain™ (Sysmex). Three randomly selected fields in each membrane were photographed under an Olympus C‐5060 light microscope (Olympus) with a 10 × objective.

### 2.13 Light microscopy

Formalin‐fixed, paraffin‐embedded sections (2‐μm thick) of primary subcutaneous tumor tissues were stained with hematoxylin and eosin. Dead cells among tumor tissues were analyzed by US National Institutes of Health Image J analysis software.

### 2.14 Tumor growth in vivo

NOD/SCID/JAK3 null female mice (8‐ to 14‐weeks old) were purchased from Kyudo Japan. Mice were subcutaneously (N = 14) or intravenously (N = 9) injected with 5 million TC71 cells. Mice were treated with 60 mg/kg TAE226 (subcutaneously 7; intravenously 4) or 200 µL of the methylcellulose as a vehicle (subcutaneously 7; intravenously 5) by daily gastric lavage 7 day after injection with TC71 cells. After the subcutaneous injection, the size of their tumors was periodically measured. Twenty‐eight days after subcutaneous injection, primary tumor volume, and weight were determined after excision at necropsy. Moreover, blood examination of five mice (TAE226 3; control 2) was performed to evaluate TAE226 toxicity. Twenty‐eight days after intravenous injection, bone marrow from tibiae was extracted and examined. The animal care and experimental procedures were conducted in strict accordance with recommendations in the Guide for the Care and Use of Laboratory Animals of the University of Miyazaki. Experimental protocols were approved by the Medicine Institutional Animal Care and Use Committee of the University of Miyazaki (Approval No. 2014‐527).

### 2.15 Bone marrow involvement by flow cytometry

Bone marrow cells were collected from femurs and tibiae. Staining of PE‐conjugated anti‐CD99 (BioLegend), APC‐conjugated anti‐CD45 (BioLegend), and APC‐conjugated Ter119 (BioLegend) was carried out at room temperature for 20 minutes. Cells were then washed in phosphate‐buffered saline at 1200 rpm for 5 minutes and resuspended in 400 µL of phosphate‐buffered saline. Samples were analyzed on a FACS Aria II cytometer (Becton Dickinson) or JSAN cell sorter (Bay Bioscience), and data were analyzed by FlowJo 8.7 software (Tree Star).

## 3 RESULTS

### 3.1 Comparison of TAE226 and PF‐562,271 antiproliferative effects on EWS cell lines

**Figure 1:**
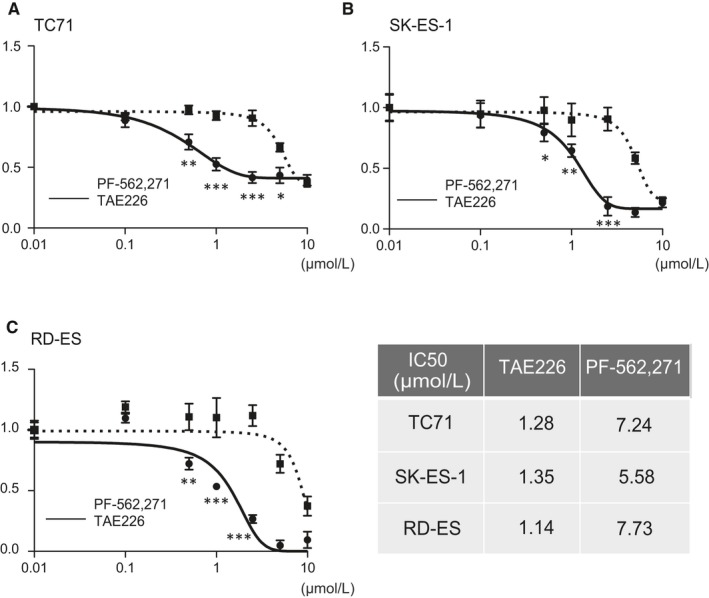
TAE226 drastically inhibits proliferation of Ewing sarcoma cell lines compared with PF‐562,271. A, TC71 cells; B, SK‐ES‐1 cells; C, RD‐ES cells. Plain and dotted lines indicate TAE226 and PF‐562,271, respectively. The data are presented as the mean ± standard deviation. IC_50_ values show that all three Ewing sarcoma cell lines were more sensitive to TAE226 than PF‐562,271. *P < .05; **P < .005; ***P < .0005. The data are presented as the mean ± standard deviation

The effects of TAE226 and PF‐562,271 on EWS cell growth were investigated with a panel of three EWS cell lines by MTT assay. All cell lines (TC71, SK‐ES‐1, and RD‐ES) were sensitive to TAE226 treatment (IC_50_ value of 1.28, 1.35, and 1.14 μmol/L, respectively), while the IC_50_ value of the most sensitive SK‐ES‐1 cells treated with PF‐562,271 was 5.58 μmol/L. These results indicate that all EWS cell lines were more sensitive to TAE226 than PF‐562,271 (Figure 1); therefore, TAE226 was chosen for all further experiments. Human fibroblasts in addition to other types of sarcoma cell lines, such as rhabdomyosarcoma (RD), osteosarcoma (HS‐Os‐1), and clear cell sarcoma (SU‐CCS‐1), were also treated with TAE226, and they definitely proved to be more resistant than EWS cells. Additionally, we compared the cytotoxicity of TAE226 and IGF‐IR (picropodophyllin) in EWS cells; TAE226 showed a stronger cytotoxic potency in EWS cells than IGF‐IR (Figure S1).

### 3.2 TAE226 effectively reduced FAK, IGF‐IR, and AKT phosphorylation in TC71 cells

**Figure 2:**
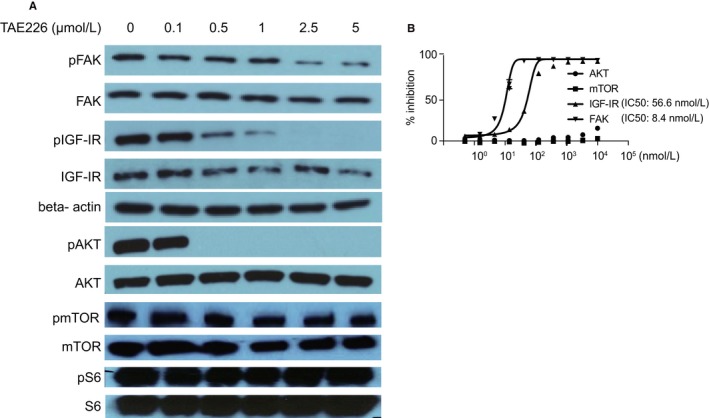
Effects of TAE226 on various pathways in TC71 cell line. A, TC71 cells were treated with various concentrations of TAE226 (0‐5 µmol/L). FAK, IGF‐IR, and AKT were dephosphorylated in a dose‐dependent manner, while the mammalian target of rapamycin (mTOR) and S6 were not. B, SelectScreen kinase profiling revealed that TAE226 selectively inhibited FAK and IGF‐IR with IC_50_ values of 8.4 and 56.6 nmol/L, respectively. However, TAE226 showed no direct effects on mTOR and AKT phosphorylation

The effect of TAE226 on FAK and IGF‐IR was investigated in TC71 cells. Phosphorylation of FAK (Tyr397) and IGF‐IR (Tyr1135/1136), which are targeted by TAE226, was significantly inhibited in a dose‐dependent manner (Figure 2A). For IGF‐IR, phosphorylation was completely inhibited by TAE226 after serum starvation regardless of ligand stimulation. Thus, TAE226 inhibited phosphorylation of IGF‐IR as expected. Next, the phosphorylation status of AKT, mTOR, and S6 was investigated after TAE226 treatment of TC71 cells. While 0.5 µmol/L TAE226 completely downregulated AKT phosphorylation, mTOR and S6 could not be dephosphorylated with 5 µmol/L TAE226 (Figure 2A).

### 3.3 TAE226 selectively inhibited FAK and IGF‐IR phosphorylation without directly inhibiting AKT and IGF‐IR phosphorylation

We performed an in vitro kinase assay with TAE226 and found that TAE226 selectively inhibited FAK and IGF‐IR with IC_50_ values of 8.4 and 56.6 nmol/L, respectively. However, mTOR and AKT phosphorylation was not inhibited, suggesting that TAE226 has no direct effect on AKT or IGF‐IR inhibition (Figure 2B).

### 3.4 Gene expression analysis

**Figure 3:**
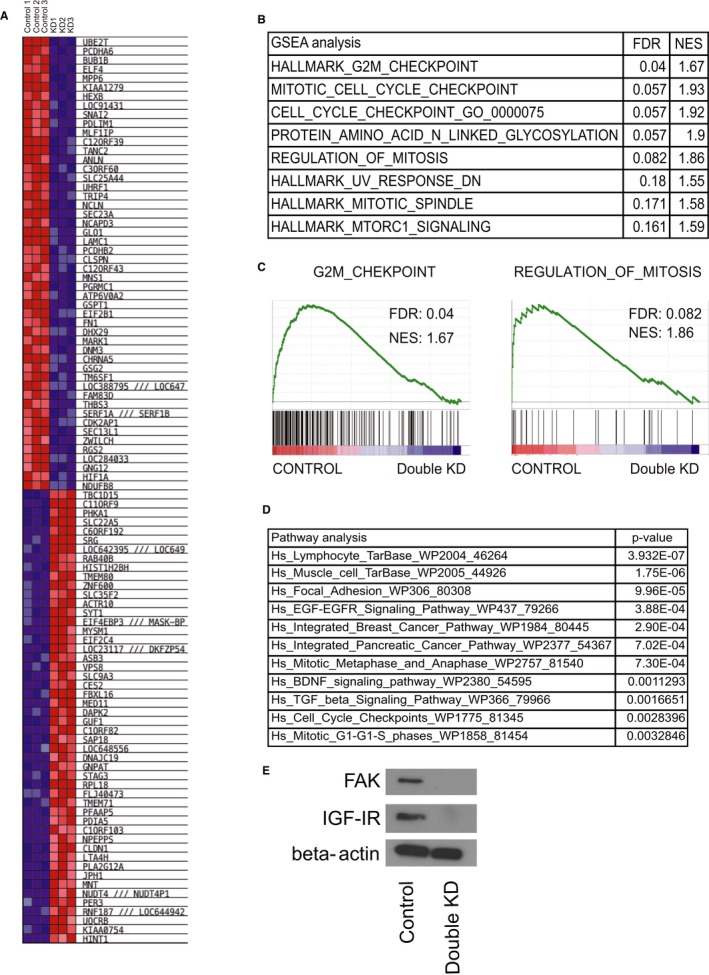
Gene expression analysis of Ewing sarcoma cells with double‐knock down of FAK and IGF‐IR. A, Gene set enrichment analysis. B and C, Enrichment for signatures associated with cell cycle checkpoints and mitosis. D, epidermal growth factor receptor (EGFR), brain‐derived neurotrophic factor (BDNF), and transforming growth factor‐β (TGF‐β) signaling pathways, as well as cell cycle checkpoints and mitotic phases were significantly altered by knockdown of FAK and IGF‐IR on pathway analysis. FDR: false discovery rate; NES: negative normalized enrichment score. E, Appropriate knockdown of FAK and IGF‐IR in the EWS cells was confirmed by western blot analysis

FAK and IGF‐IR are therapeutic targets in EWS; however, which signaling pathway is regulated in TC71 cells when both are simultaneously inhibited has not been clarified. To determine the effect of FAK and IGF‐IR pathway inhibition, we first analyzed genes modulated upon FAK and IGF‐IR knockdown using gene set enrichment analysis (Figure 3A), and enrichment of signatures associated with cell cycle checkpoints and mitosis were found (Figure 3B,C). EGFR, BDNF, and TGF‐β signaling pathways were also significantly altered by knockdown of FAK and IGF‐IR according to pathway analysis (Figure 3D and Table S1). These results suggested that dual inhibition of FAK and IGF‐IR not only induced an antiproliferative effect by cell cycle regulation but also through signals involved in cellular infiltration and metastasis. Appropriate knockdown of FAK and IGF‐IR in the EWS cells was confirmed by RQ‐RT‐PCR and western blot analysis (Figure 3E). In addition, we confirmed the same gene expression pattern in TC71 cells treated with 10 μmol/L of TAE226 for 6 hours. BDNF, EGFR, and TGF‐β signaling pathways as well as cell cycle‐ and apoptosis‐related genes were modulated in a manner consistent with the result of the knockdown experiments (Table S2). Furthermore, we analyzed the AKT phosphorylation status after stimulating TC71 cells with EGF, BDNF, or TGF‐β in the presence of TAE226 treatment to assess whether TAE226 suppresses EGFR, BDNF, and TGF‐β signaling pathways. TAE226 downregulated AKT phosphorylation following BDNF, EGF, and TGF‐β stimulations, although the levels of AKT phosphorylation were varied, suggesting that TAE226 suppresses the BDNF, EGFR, and TGF‐β signaling pathways (Figure S2).

### 3.5 Low concentration of TAE226 induced cell cycle arrest through the reduction of S phase in EWS cell lines

**Figure 4:**
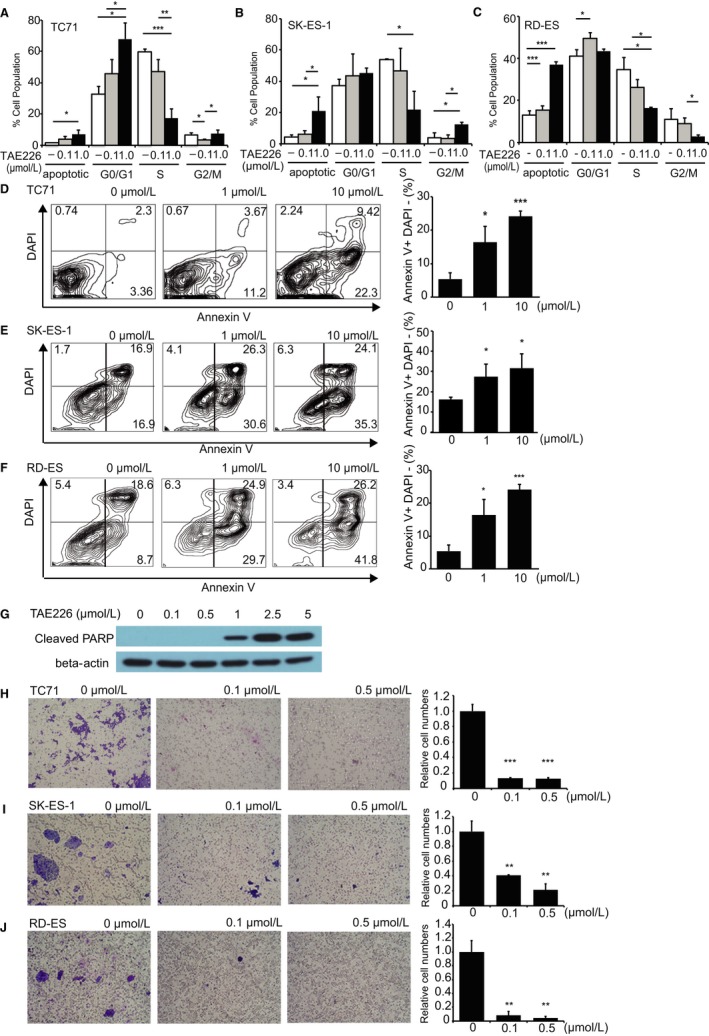
High concentration TAE226 induced apoptosis, while low concentration TAE226 inhibited invasion and cell cycle arrest in Ewing sarcoma cell lines. A‐C, Cell cycle parameters in TC71 (A), SK‐ES‐1 (B), and RD‐ES (C) cells treated with 0.1 and 1.0 µmol/L TAE226. The population of S‐phase cells decreased together with an increase in the population of the G0/G1‐ (TC71: A) and G2/M‐phase cells (SK‐ES‐1: B) and a drastic increase in the population of apoptotic cells (RD‐ES: C). D‐F, TC71 (D), SK‐ES‐1 (E), and RD‐ES (F) cells treated with 1 or 10 µmol/L TAE226 were analyzed by flow cytometry after staining with APC Annexin‐V and 4',6‐diamidino‐2‐phenylindole dihydrochloride (DAPI). The number of apoptotic cells significantly increased in a concentration‐dependent manner. G, Poly‐ADP‐ribose polymerase (PARP) cleavage occurred with 1 µmol/L TAE226 treatment. H‐J, Cellular Matrigel™ invasion by TC71 (H), SK‐ES‐1 (I), and RD‐ES (J) cells decreased after treatment with 0.1 or 0.5 µmol/L TAE226. TAE226 strongly inhibited invasion by both cell types. *P < .05; **P < .005; ***P < .0005. The data are presented as the mean ± standard deviation

Since pathway analysis revealed altered expression of genes involved in the cell cycle, cell cycle parameters were investigated in EWS cell lines treated with a low concentration of TAE226 because cell death was induced at a high concentration. Even at a low concentration, the sub G1 populations (ie, apoptotic fraction) in all the three cell lines were significantly increased in a concentration‐dependent manner. The results showed that the population of S‐phase cells in all the three cell lines decreased together with an increase in the population of the G0/G1‐ (TC71: Figure 4A) and G2/M‐phase cells (SK‐ES‐1: Figure 4B) and a drastic increase in the population of apoptotic cells (RD‐ES: Figure 4C).

### 3.6 High concentration TAE226 induced apoptosis in EWS cell lines

To confirm induction of apoptotic cell death by TAE226, EWS cells treated with 1 or 10 µmol/L TAE226 were analyzed by flow cytometry after staining with APC Annexin‐V and 4',6‐diamidino‐2‐phenylindole dihydrochloride. The population of apoptotic cells was significantly increased in a concentration‐dependent manner (Figure 4D‐F). Western blot analysis revealed cleaved PARP was introduced after 1 µmol/L TAE226 treatment (Figure 4G).

### 3.7 Low concentration of TAE226 inhibited invasion in EWS cell lines

Since EGFR and TGF‐β signaling pathways involved in cell movement and metastasis were found to be altered in gene expression analysis (Figure 3D), we explored the potential for TAE226 to inhibit tumor invasion and metastasis of EWS cells. Cellular invasion of Matrigel™ was found to be decreased with 0.1 or 0.5 µmol/L TAE226 treatment (Figure 4H‐J); TAE226 also inhibited cellular invasion.

### 3.8 Antitumor effect of TAE226 on mouse EWS xenotransplantation models

**Figure 5:**
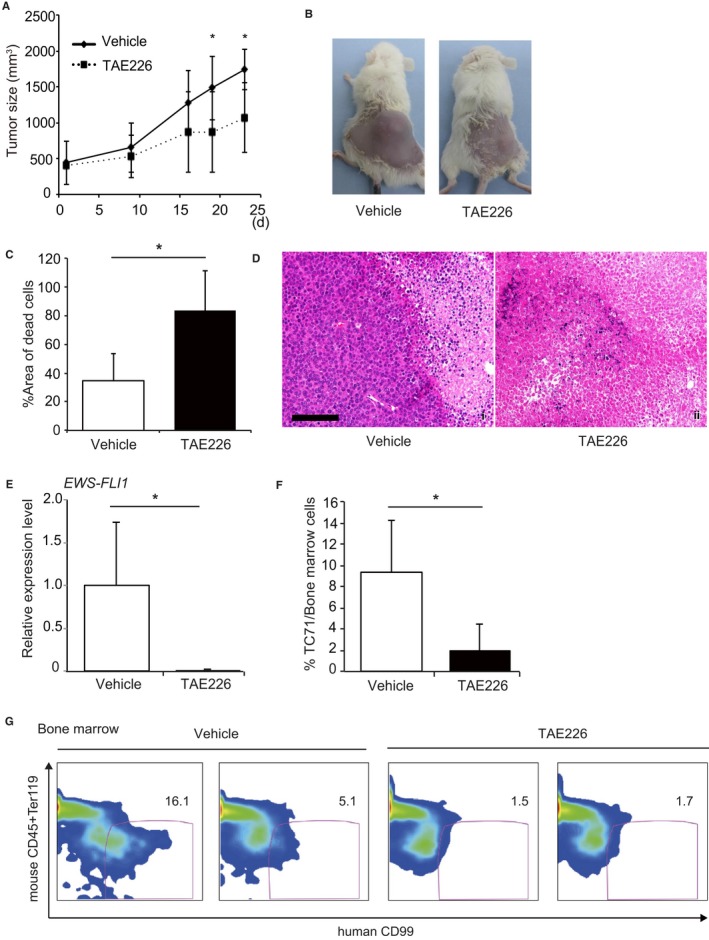
In vivo effect of TAE226 on TC71 cells after subcutaneous and intravenous xenotransplantation. A‐D, Suppression of primary tumor growth in subcutaneous xenotransplantation mice by TAE226 oral administration. Seven days after TC71 cells were subcutaneously injected, TAE226 (60 mg/kg) or methylcellulose (vehicle) was orally administered for 21 d (days 7‐28). After the subcutaneous injection, the size of their tumors was periodically measured (A). Tumor size was reduced by TAE226 treatment (A, B), and dead cells were predominant in tumor tissues (C). Images from a typical experiment are presented (B, D). Formalin‐fixed, paraffin‐embedded sections of primary subcutaneous tumor tissues were stained with hematoxylin and eosin. Scale bars, 100 µm (D). E‐G, Suppression of bone marrow metastasis of intravenously xenotransplanted mice by TAE226 oral administration. Seven days after TC71 cells were intravenously injected, TAE226 (60 mg/kg) or methylcellulose (vehicle) was orally administered for 21 d. On day 28, bone marrow cells were collected from femurs and tibiae and subjected to RQ‐RT‐PCR assessment of the *ESW‐FLI1* transcript (E) and flow cytometric analysis with an anti‐CD99 antibody (F). Decreased tumor involvement was observed in both analyses (E. F). Representative flow cytometry data are shown (G). Error bars represent standard deviation. The data are presented as the mean ± standard deviation; *P < .05

The inhibitory effect of TAE226 on proliferation and metastasis in vivo was analyzed by subcutaneous and intravenous injection of TC71 cells in mice, respectively. Three weeks of TAE226 treatment after 1‐week interval from subcutaneous injection of TC71 cells apparently reduced the size and weight of the tumors (Figure 5A,B). The percentage of dead cells in subcutaneous tumors of mice treated with TAE226 were significantly higher than untreated control mice (Figure 5C,D). Blood examination indicated no drastic difference in terms of renal and hepatic function between TAE‐treated and non‐treated mice, although TAE226‐treated mice presented with leukopenia and anemia (Table S3). In TAE226 treatment for 3 weeks after 1‐week interval from intravenous injection of TC71 cells, the *EWS‐FLI‐1* transcript was significantly decreased in the bone marrow of the mice with TAE226 treatment vs. control mice (Figure 5E). We also confirmed striking decreased EWS‐specific anti‐human CD99 positive cell population in the bone marrow (Figure 5F,G). These data showed that TAE226 drastically decreased metastatic potential as well as local growth of EWS cells in vivo.

### 3.9 Anticancer drugs had synergistic effect with TAE226 on EWS cells

Three EWS cell lines (TC71, SK‐ES‐1, and RD‐ES) were treated for 48 hours with a combination of TAE226 and other anticancer drugs (vincristine, doxorubicin, and etoposide). Cell viability was analyzed by MTT assay. The potency of each drug combination was quantified using CalcuSyn software (Bio‐soft Inc), which is based on the Chou‐Talalay method, yielding a combination index (CI) with the following interpretation: CI > 1, antagonistic effect; CI = 1, additive effect; CI < 1, synergistic effect. Combining each of the three chemotherapeutics at various ratios showed synergistic effects (<1) with low TAE226 concentrations (0.1 and 0.5 µmol/L; Table 1 and Figure S3).

**Table 1:** Combination index for TAE226 and chemotherapeutics

Cell line	Anticancer drugs	Ratio (TAE226: each drug)	ED50	ED75	ED90
TC71	Doxorubicin	1:0.1	0.72363	0.88349	1.4976
	Vincristine	1:0.001	0.39522	0.51643	0.6931
	Etoposide	1:0.5	0.85054	17.11703	492.47009
		1:1	1.91545	5452.53666	2.04E + 07
SK‐ES‐1	Doxorubicin	1:0.1	0.4115	0.65838	1.05355
	Vincristine	1:0.001	0.56041	0.81403	1.18253
	Etoposide	1:0.5	0.44542	0.96267	2.08094
		1:1	0.01927	56.267	1.64E + 05
RD‐ES	Doxorubicin	1:0.1	0.34303	0.41376	0.49908
	Vincristine	1:0.001	0.32721	0.68391	1.42971
	Etoposide	1:0.5	0.29148	0.52327	0.93956
		1:1	0.37823	0.40577	0.4355

Note

The potency of each drug combination was quantified with CalcuSyn software. A combination index (CI) with the following interpretation: CI > 1, antagonistic effect; CI = 1, additive effect; CI < 1, synergistic effect (filled with green color). Combining each of the three chemotherapeutics at various ratios showed synergistic effects (<1) with low TAE226 concentrations.

## 4 DISCUSSION

The prognosis for localized EWS without metastasis has been improved with conventional chemotherapy combined with surgery or radiotherapy for local control. However, outcomes for relapsed and metastatic forms of the disease are extremely poor.^3^, ^4^, ^28^ Therefore, it is important to identify tumor‐specific targets in these intractable diseases. FAK is an attractive candidate target for EWS, and several small molecules that inhibit FAK activity have already been developed.

In the present study, we first compared the anti‐EWS effect between two small molecules, TAE226 (FAK and IGF‐IR dual inhibitor) and PF‐562,271 (FAK and PYK2 dual inhibitor), and found that all EWS cell lines tested were more sensitive to TAE226. PYK2 is an FAK‐related family member that shares 65% amino acid sequence similarity and shows a similar effect to FAK in regulation of cancer cell mortality and invasion.^29^, ^30^ IGF‐IR is a tyrosine kinase receptor that activates both mitogenic and antiapoptotic pathways after binding its ligands, IGF‐I or IGF‐II.^31^ Mesenchymal cells transformed by EWS‐FLI1 have been shown to express increased levels of IGF‐I and depend on IGF‐IR signaling for growth and survival.^32^ Similarly, IGF‐IR expression is required for EWS‐FLI1‐mediated transformation in murine fibroblasts.^33^ A clinical study with the IGF‐IR antibody has demonstrated its meaningful and durable benefit in a subgroup of patients with EWS.^10^ Hence, our current results demonstrating the superiority of TAE226 over PF‐562,271 in EWS is reasonable, although whether IGF‐IR inhibitor is superior to PYK2 inhibitor remains uncertain.

Next, we established a stable TC71 cell line with knocked down FAK and IGF‐IR expression targeting shRNA to clarify which pathways are concerned using microarray. The results revealed differential expression of genes related to cell cycle checkpoints and mitosis, as well as EGFR, BDNF, and TGF‐β signaling in mutant samples compared to controls. Western blotting results proved that BDNF, EGFR, and TGF‐β signaling pathways are related to TAE226 treatment. Flow cytometric analysis showed that low and high concentrations of TAE226 induced cell cycle arrest and apoptosis, respectively. Furthermore, western blot was used to investigate protein expression patterns with TAE226 treatment. FAK is known as an upstream regulator of AKT in normal tissue and models of cancer.^34^, ^35^ The mTOR pathway is activated in a subset of EWS.^36^, ^37^, ^38^ Previous studies have shown that inhibition of IGF‐IR leads to downregulation of AKT and mTOR phosphorylation in EWS.^39^ Our results showed the dual FAK and IGF‐IR inhibitor TAE226 downregulated AKT phosphorylation but not mTOR. This suggests another mechanism exists in overcoming mTOR downregulation by dephosphorylation of FAK and IGF‐IR in EWS. Complete AKT dephosphorylation was observed at a TAE226 concentration of 0.5 µmol/L, suggesting that TAE226 may directly inhibit AKT phosphorylation. However, TAE226 showed no direct effect on AKT phosphorylation in the in vitro kinase assay, and the underlying mechanism of this phenomenon has not been elucidated.

Finally, we explored the antitumor effect of TAE226 on mouse xenotransplantation models. Our in vitro and in vivo studies show that EWS is dependent on FAK and IGF‐IR activity for growth, invasion, progression, and metastasis. In particular, current in vivo results on bone marrow metastasis with TAE226 treatment are important to the development of a clinical trial implementing this compound. Several clinical trials using FAK inhibitors have already been carried out in advanced solid tumors.^40^, ^41^, ^42^ Interestingly, several patients have response based on evaluation of positron emission tomography and prolonged stabilization of disease by treatment with PF‐562,271.^40^ Herein, we also found that combined TAE226 and conventional chemotherapeutic (vincristine, doxorubicin, and etoposide) treatment had synergistic effects against EWS. Recently, other candidate EWS inhibitors, such as EGFR and Trk inhibitors,^43^, ^44^ have been reported to be potential future therapeutic agents. A combination therapy of these drugs in addition to conventional chemotherapy and TAE226 treatment may improve the prognosis of patients with EWS.

In conclusion, systemic TAE226 treatment potently reduced the size of local tumors and inhibited micrometastasis in vivo through cell cycle inhibition, induction of apoptosis, and inhibition of AKT signaling. Furthermore, combined therapy with TAE226 and conventional anticancer drugs for EWS has synergistic anticancer effects. Overall, the results of the present study suggest that TAE226 is a candidate single agent or combined therapy drug to be developed for patients who have relapse and metastatic EWS tumors in future.

## CONFLICTS OF INTEREST

The authors declare that they have no conflicts of interest in association with this study.
