# Supplementary material for: TAE226, a dual inhibitor of focal adhesion kinase and insulin‐like growth factor‐I receptor, is effective for Ewing sarcoma
Source: Cancer Med. 2019 Nov 6;8(18):7809–21. doi: 10.1002/cam4.2647 (PMC6912025; doi:10.1002/cam4.2647)
Supplement: Supplementary file 1 [file CAM4-8-7809-s001.pdf]

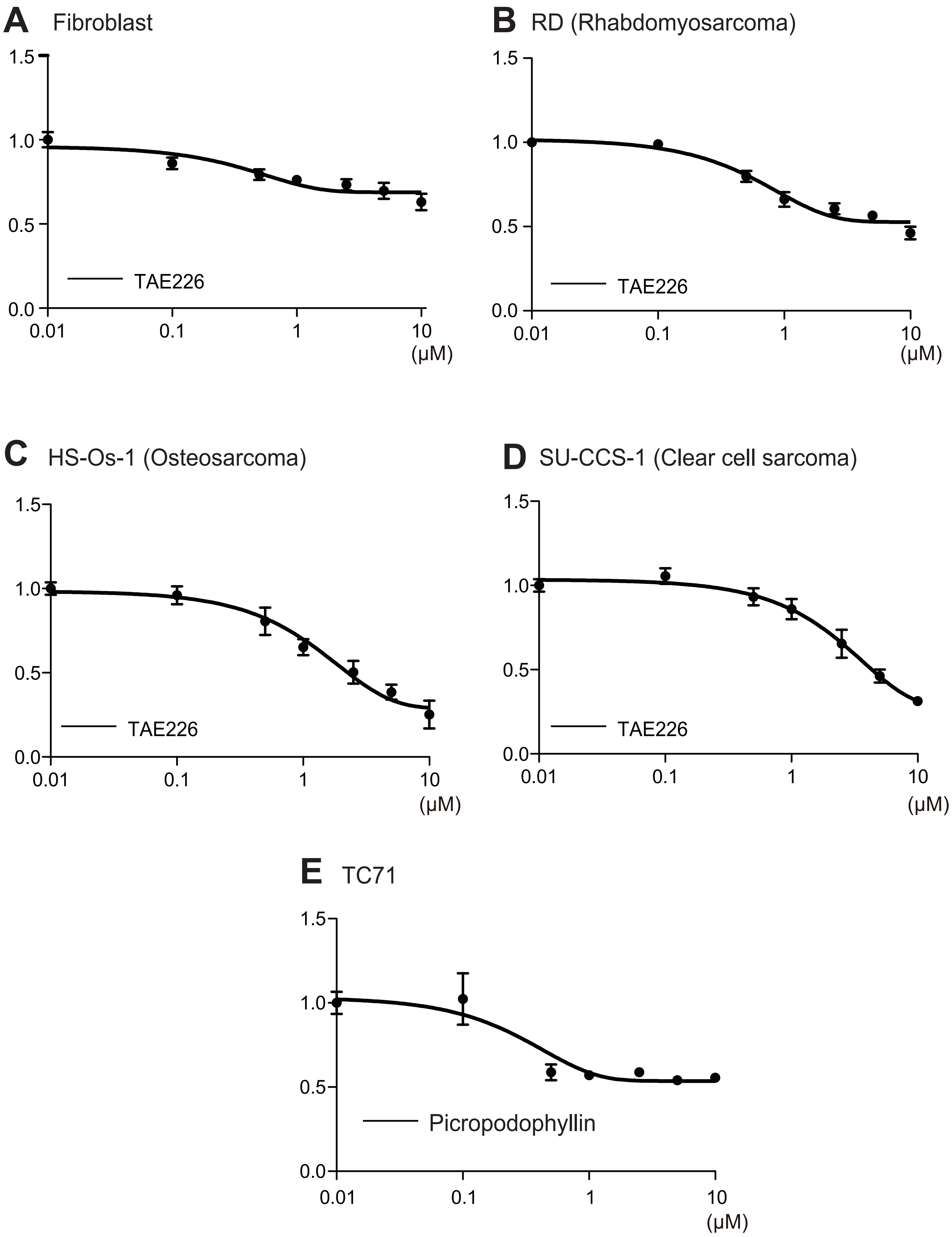

Figure S1. Human fibroblasts (A), rhabdomyosarcoma (B), osteosarcoma (C), and clear cell sarcoma (D) treated with TAE226 definitely proved to be more resistant than Ewing sarcoma cells (Figure 1). TAE226 showed a stronger cytotoxic potency in Ewing sarcoma cells than IGF-IR inhibitor (Picropodophyllin) (E).
