# Supplementary material for: TAE226, a dual inhibitor of focal adhesion kinase and insulin‐like growth factor‐I receptor, is effective for Ewing sarcoma
Source: Cancer Med. 2019 Nov 6;8(18):7809–21. doi: 10.1002/cam4.2647 (PMC6912025; doi:10.1002/cam4.2647)
Supplement: Supplementary file 2 [file CAM4-8-7809-s002.pdf]

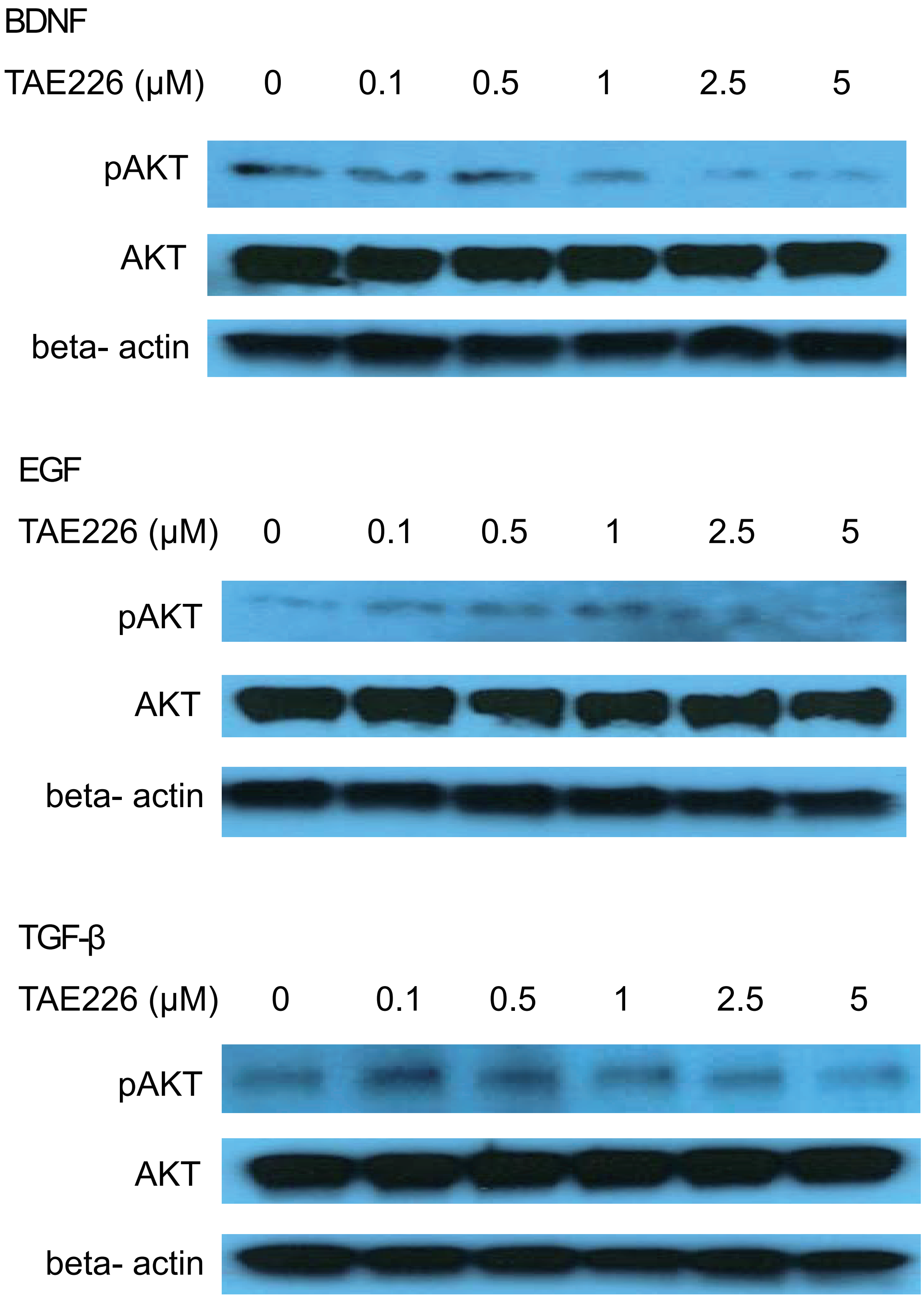

Figure S2. TAE226 downregulated the phosphorylation of AKT after BDNF, EGF and TGF-β stimulations, although the levels of AKT phosphorylation were varied, suggesting that TAE226 suppresses the BDNF, EGFR, and TGF-β signaling pathways.
