# Supplementary material for: TAE226, a dual inhibitor of focal adhesion kinase and insulin‐like growth factor‐I receptor, is effective for Ewing sarcoma
Source: Cancer Med. 2019 Nov 6;8(18):7809–21. doi: 10.1002/cam4.2647 (PMC6912025; doi:10.1002/cam4.2647)
Supplement: Supplementary file 3 [file CAM4-8-7809-s003.pdf]

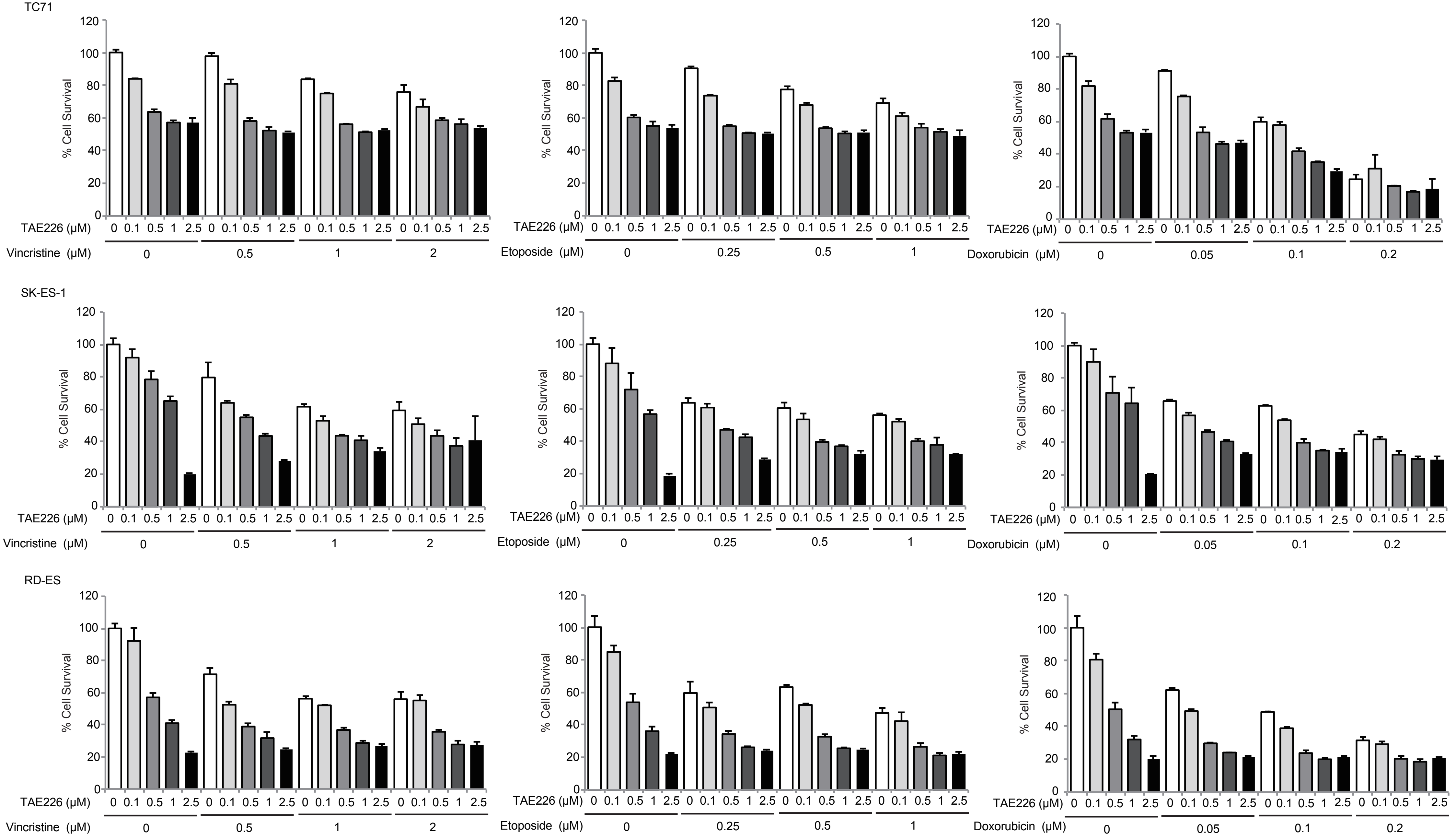

Figure S3. TAE226 and anticancer drugs have synergistic effects against Ewing sarcoma cells.
Vincristine, doxorubicin, and etoposide are cytotoxic to Ewing sarcoma cells in a dose-dependent manner when co-administered with various concentrations of TAE226 according to MTT assay results. These effects were confirmed by CalcuSyn software.
